# Supplementary material for: pyTFM: A tool for traction force and monolayer stress microscopy
Source: PLoS Comput Biol. 2021 Jun 21;17(6):e1008364. doi: 10.1371/journal.pcbi.1008364 (PMC8248623; doi:10.1371/journal.pcbi.1008364)
Supplement: S1 Text — First, we discuss the definition and interpretation of the quantities that pyTFM uses to describe cellular stresses, force generation and cell-cell force transfer. Next, we analyze the effect of FEM-element size on the stress calculation. (PDF) [file pcbi.1008364.s001.pdf]

1 Stress and force generation

pyTFM uses the deformation field of the substrate to calculate the traction and stress fields and from them a number of scalar parameters that characterize contractile force generation, intra- and inter-cellular stress levels and stress distribution.

1.1 Strain energy and contractility

Contractile forces can be characterized by the strain energy and the contractility, both of which are evaluated across a user-selected area that contains all cell-generated tractions (red outline in Fig. 5). The strain energy is the elastically stored energy in the substrate. It is calculate according to:

$$U = \frac{1}{2}\sum_{\Omega}(\vec{t} * A_p * \vec{u}) - U_{bg} \tag{1}$$

where $\vec{t}$, and $\vec{u}$ are traction and deformation vectors, A_p is the area of a pixel and $\sum_\Omega$ indicates the summation over the user selected area around the cells. U_{bg} is a correction term that accounts for background noise. It is obtained from the 20-th percentile of strain energies per pixel of the entire field of view.

The strain energy is not sensitive to the spatial organization of tractions. By contrast, the contractility characterizes not only the magnitude but also the spatial coordination of tractions; it is computed according to [1] as the sum of all traction vectors that are oriented towards a force "epicenter" (Fig. S1). The force epicenter is defined as the point where the sum of projected tractions reaches a maximum.

Note that the contractility, in contrast to the strain energy, does not require a background correction as the projected background tractions are randomly oriented and therefore sum up to zero.

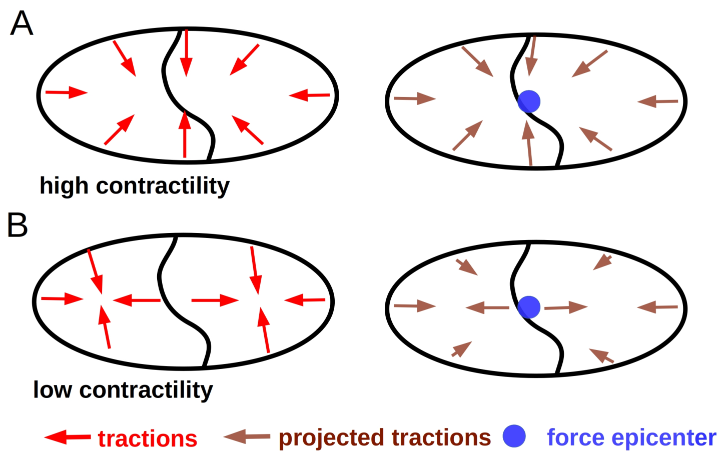

Fig S1. Contractility as a measure for coordinated force generation. Highly coordinated force generation (A) is characterized by the orientation of all traction vectors towards a force epicenter. Consequently, the contractility (the sum of the projections of the traction vectors towards this epicenter) is large. Cells with weakly coordinated force generation (B) appear to have no single discernible force epicenter. In this case, tractions parallel to the global force epicenter contribute little to the contractility, and tractions pointing away from the global force epicenter reduce the contractility.

1.2 Stresses in cells and cell patches

Stress describes the force-per-area (i.e. the traction) acting on an imaginary cross-section through the material, which depends on the orientation of the cross-sectional plane. This relationship is described by Cauchy's stress theorem [2]:

$$\vec{t} = \sigma * \vec{n} \tag{2}$$

whereby $\vec{t}$ is the traction vector, $\vec{n}$ is the vector normal to the cross-sectional plane, and σ is the stress tensor. Since we model the cells as 2-dimensional materials, the cross-sectional planes are replaced by cross-sectional lines, and the traction vector $\vec{t}$ becomes a line tension vector in units of force-per-length.

The 2-dimensional stress tensor, illustrated in Fig. S2, has 4 components. The first column of the stress tensor corresponds to the line tension vector acting on the cross-sectional line parallel to the y-axis, whereby σ_{xx} and σ_{xy} are the normal and shear component of the line tension vector. Similarly, σ_{yy} and σ_{yx} are the normal and shear

components of the line tension vector acting on the cross-sectional line parallel to the x-axis. pyTFM models the cell layer as an isotropic material, meaning that the material has the same elastic properties in all directions. In such a material, the shear components σ_{xy} and σ_{yx} are identical.

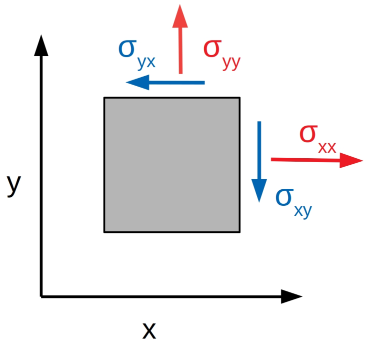

$$\sigma = \begin{bmatrix} \sigma_{xx} & \sigma_{xy} \\ \sigma_{yx} & \sigma_{yy} \end{bmatrix}$$

Fig S2. Stresses in a 2-dimensional material- The components of the stress tensor σ_{xx} and σ_{xy} are the normal and shear component of the force vector per unit line length acting on an infinitesimal small cross-sectional line that is oriented parallel to the y axis. σ_{yy} and σ_{xy} are the normal and shear component of the force vector per unit line length acting on an infinitesimal small cross-sectional line that is oriented parallel to the x axis.

Because the orientation of the coordinate system is arbitrarily chosen, the line tensions $\vec{t}$ at the cross-sectional lines parallel to the x- and y-axis are of no particular interest. Instead, it is more instructive to analyze the set of all possible cross-sectional lines and to compute the maximum and the average line tension, and the variation (or "anisotropy") of the line tensions. The magnitudes of the maximum and the minimum line tension vectors correspond to the so called principal stresses σ_{max} and σ_{min}. They are the eigenvalues of the stress tensor and can be calculated according to:

$$\begin{aligned} \sigma_{max} &= \frac{\sigma_{xx} + \sigma_{yy}}{2} + \sqrt{\frac{\sigma_{xx} + \sigma_{yy}}{2} - \sigma_{xy} **2} \\ \sigma_{min} &= \frac{\sigma_{xx} + \sigma_{yy}}{2} - \sqrt{\frac{\sigma_{xx} + \sigma_{yy}}{2} - \sigma_{xy} **2} \end{aligned} \tag{3}$$

The average line tension is defined as the average normal component of the line tension σ_n (also called the mean normal stress), which can be calculated simply by averaging σ_{max} and σ_{min}:

$$\sigma_n = \frac{\sigma_{max} + \sigma_{min}}{2} \tag{4}$$

Finally, the variation of the line tension magnitudes is best described by the maximum shear stress σ_{s_max}, which is simply the difference between σ_{max} and σ_{min} divided by 2:

$$\sigma_{s\_max} = \frac{\sigma_{max} - \sigma_{min}}{2} \tag{5}$$

Note that the minimum shear stress σ_{s_min} is simply the negative of the maximum shear stress.

pyTFM averages the mean normal stresses over the user-selected area of the cell colony ($\overline{\sigma}_n$). $\overline{\sigma}_n$ can have a positive sign, indicating that tensile stresses dominate throughout the cell colony, or a negative sign, indicating that compressive stresses dominate throughout the cell colony. A typical cell colony generates forces that pull the surrounding matrix inwards and that need to be balanced by tensile stresses - in this case, $\overline{\sigma}_n$ has a positive sign.

pyTFM also averages the maximum normal stresses ($\overline{\sigma}_{max}$) and the maximum shear stresses ($\overline{\sigma}_{s\_max}$) over the area of the cell colony. $\overline{\sigma}_{s\_max}$ and $\overline{\sigma}_{max}$ can only assume positive values; $\overline{\sigma}_{max}$ characterizes the overall stress levels, and $\overline{\sigma}_{s\_max}$ characterizes the local stress anisotropy averaged over the cell colony.

1.3 Stress distribution in cells and cell patches

Analogous to the average stresses explained above, pyTFM also quantifies how evenly stresses are distributed across the cell patch by calculating the coefficient of variation (CV) for the maximum shear stress (σ_{s_max}), the maximum normal stress, and the mean normal stress (σ_n) (σ_{max}):

$$CV = \frac{\sqrt{\frac{1}{N}\sum_{\Omega}(\sigma_i - \bar{\sigma_i})^2}}{\bar{\sigma_i}} \tag{6}$$

where σ_i is one of the stresses mentioned above, and $\overline{\sigma_i}$ is their average computed over the cell patch area Ω. The coefficient of variation tends to zero for a completely uniform stress field.

1.4 Cell-cell force transduction in cell patches

Cell-cell force transduction is quantified by the line tension at the cell-cell boundaries. As mentioned above, the stress tensor can be used to calculate forces-per-length acting across arbitrarily oriented cross-sectional lines. If the cross-sectional line follows the cell-cell boundary, the line tension at this point corresponds to the force transmitted between cells. In practice, the line tension is calculated as follows: The user marks all cell boundaries by drawing a mask (with the brush tool) in the *ClickPoints* environment. The mask is then reduced to one-pixel wide lines using the *skimage* skeletonize function. Next, the lines are smoothed by a 2-dimensional spline interpolation. Cell boundaries at the colony edge are identified, and the line tension at all internal cell-cell boundaries is evaluated. This is done by taking small (sub-pixel long) line segments and applying Eq. 2. The normal vector $\vec{n}$ is obtained from the derivative of the spline representation of the small line segment. The resulting line tension vector $\vec{l_t}$ has x and y components, however, analogous to the discussion above, it is more instructive to describe the line tension by its magnitude l_{tm}, normal l_{tn} and shear l_{ts} components. These quantities are calculated according to:

$$
\begin{aligned}
l_{tm} &= ||\vec{l_t}|| \\
l_{tn} &= \vec{l_t} * \vec{n} \\
l_{ts} &= \sqrt{l_{tm}^2 - l_{tn}^2}
\end{aligned}
\tag{7}
$$

and subsequently averaged over all cell boundaries.

2 Effect of FEM-element size on the accuracy of stress calculation

The FEM-element size can have a significant effect on the accuracy of the calculated stresses. Elements that are too large cannot adequately describe the usually irregular shape of the cell or cell patch. Moreover, large elements smooth over local fluctuations of cell tractions and stresses, which will tend to result in an underestimation of the true stresses. In the following, we estimate the influence of the element size on the calculated stresses in an MDCK cell colony. The edge length of the quadrilateral FEM-elements in

pyTFM is identical to the pixel size of the traction and deformation field and can be controlled by the overlap of the PIV correlation windows. We first calculate a deformation and traction field with a pixel size of 0.8 µm. Then we use spline interpolation to obtain traction fields with larger pixel sizes and use the interpolated traction fields to calculate stress fields. Finally, we compare average normal and shear stresses of the calculated stress fields (Fig. S3).

We find that the calculated stresses are nearly constant up to a FEM-element size of 3 µm but decrease for larger element sizes. 3 µm corresponds to an overlap of the PIV correlation windows of 17 µm when using the default PIV correlation window size of 20 µm. The default parameters of pyTFM (”PIV overlap” of 19 µm and “PIV windowsize” of 20 µm) correspond to an element size of 1 µm, which is well within the plateau of calculated stresses. Lowering the grid size further would only add unnecessary computational cost.

The overlap and size of the PIV correlation windows (controlled by the parameters “PIV overlap” and “PIV windowsize”) are chosen by the user based on the specific experimental setup. More detailed instructions on choosing these parameters are given in the pyTFM documentation at `https://pytfm.readthedocs.io/`.

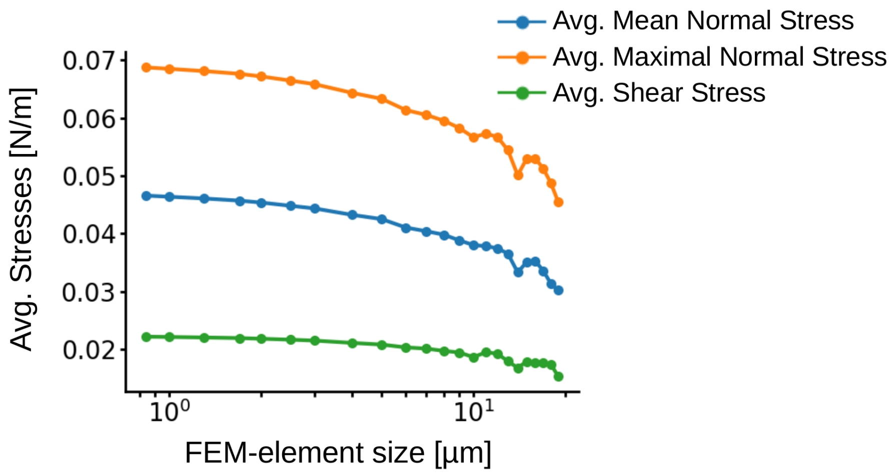

Fig S3. Effect of FEM-element size on the accuracy of stress recovery.

References

1. Steinwachs J, Metzner C, Skodzek K, Lang N, Thievessen I, Mark C, et al. Three-dimensional force microscopy of cells in biopolymer networks. Nature Methods. 2015;13(2):171–176. doi:10.1038/nmeth.3685.

2. Nair S. Introduction to continuum mechanics. New York, NY: Cambridge University Press; 2009.
