# Supplementary material for: pyTFM: A tool for traction force and monolayer stress microscopy
Source: PLoS Comput Biol. 2021 Jun 21;17(6):e1008364. doi: 10.1371/journal.pcbi.1008364 (PMC8248623; doi:10.1371/journal.pcbi.1008364)
Supplement: S2 Text — We provide basic information on our protocols for polyacrylamide gel preparation and cell culture for the TFM analysis of the MDCK cell colony. (PDF) [file pcbi.1008364.s002.pdf]

1 Cell culture and experimental procedure

Elastic polyacrylamide-bis-acrylamide (PAA) hydrogels for traction microscopy (7,85% (w/v) acrylamide and 0.27% (w/v) bis-acrylamide) were prepared according to [1]. The Young's modulus and of the gels was 18.5 kPa [2].

Green fluorescent carboxylate-modified 1 µm beads (Invitrogen) were suspended in the polyacrylamide mixture (5 µl in 500 µl), APS and TEMED was added and 28 µl of solution was pipetted to each 25 µl Gene Frame (Thermo Scientific) and covered with activated coverslips. To form a uniform bead layer on the top of the PAA gels, Gene Frames with PAA mixture were centrifuged at 300 g for 30 min at 4 °C prior the gel polymerisation. Polymerised gels were stuck at the bottom of 35 mm Petri dishes with 13 mm holes (Cell EG) [3]. The gel surface was activated with0.5 mg/ml sulfo-SANPAH (Thermo Scientific) and coated with 50 µg/ml collagen I (Gibco).

MDCK cells were cultured in DMEM media (Sigma-Aldrich) supplemented with 10% foetal bovine serum (Gibco) and 1% penicillin/streptomycin (Sigma-Aldrich) in a cell culture incubator (5% CO2, 37 °C). 30 000 MDCK cells were seeded in each 35 mm dish 48 h prior to imaging, to allow for the formation of cell colonies. Imaging was performed using Dragonfly spinning disc confocal microscope (Andor) equipped with a 20x water-immersion objective, Zyla sCMOS camera with 1.5x zoom and climate chamber (Okolab). Images were acquired using Fusion software (Andor). For each field of view with cell colony a set of three images was taken: 1) Image of cell membranes (facilitated either by a tdTomato-farnesyl membrane tag). 2) Image of fluorescent beads underneath cell colonies and 3) Image of fluorescent beads after cell detachment with Trypsin/EDTA (0.5/0.02%) solution (Sigma) for 20 minutes. The images of beads in the tensed and relaxed state where analyzed in pyTFM with the following analysis parameters:

Table 1. pyTFM analysis parameters for the analysis of a MDCK cell colony.

Young's modulus	18.5 kPa
Poisson's ratio	0.49
PIV window size	20 µm
PIV overlap	19 µm
pixel size	0.201 µm
gel height	300 µm

References

1. Pelham RJ, l Wang Y. Cell locomotion and focal adhesions are regulated by substrate flexibility. Proceedings of the National Academy of Sciences. 1997;94(25):13661–13665. doi:10.1073/pnas.94.25.13661.

2. Yeung T, Georges PC, Flanagan LA, Marg B, Ortiz M, Funaki M, et al. Effects of substrate stiffness on cell morphology, cytoskeletal structure, and adhesion. Cell Motility and the Cytoskeleton. 2004;60(1):24–34. doi:10.1002/cm.20041.

3. Mierke CT, Kollmannsberger P, Zitterbart DP, Smith J, Fabry B, Goldmann WH. Mechano-Coupling and Regulation of Contractility by the Vinculin Tail Domain. Biophysical Journal. 2008;94(2):661–670. doi:10.1529/biophysj.107.108472.
