# Supplementary material for: pyTFM: A tool for traction force and monolayer stress microscopy
Source: PLoS Comput Biol. 2021 Jun 21;17(6):e1008364. doi: 10.1371/journal.pcbi.1008364 (PMC8248623; doi:10.1371/journal.pcbi.1008364)
Supplement: S1 Archive — This archive contains the pyTFM source code and documentation which includes installation and usage instructions and links to further example data sets. (ZIP) [file pcbi.1008364.s004.zip › pyTFM/pyTFM/TFM_addon/Desc.html]

Measurement of force generation and stresses in cells and cell sheets.
