# Supplementary figures and images for: pyTFM: A tool for traction force and monolayer stress microscopy
Source: PLoS Comput Biol. 2021 Jun 21;17(6):e1008364. doi: 10.1371/journal.pcbi.1008364 (PMC8248623; doi:10.1371/journal.pcbi.1008364)

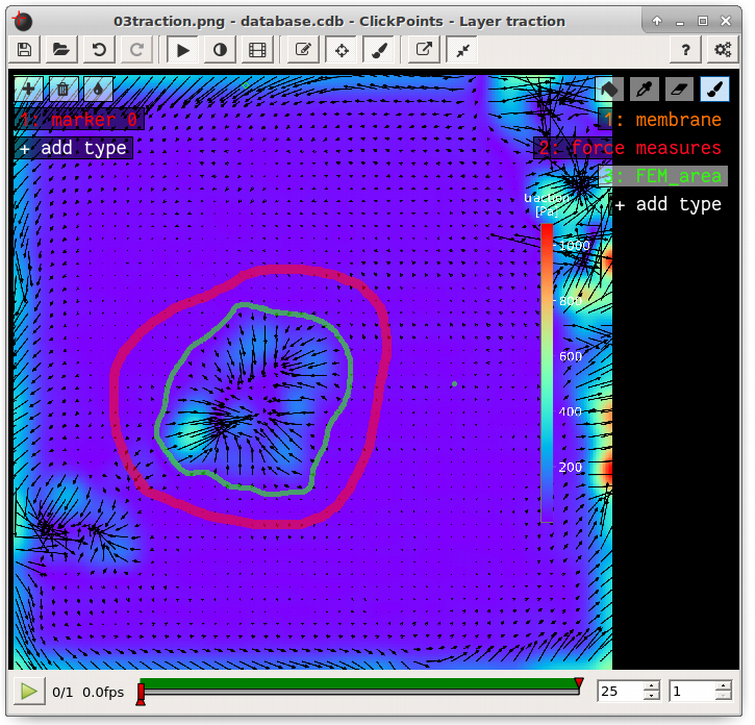

Supplement: S1 Archive — This archive contains the pyTFM source code and documentation which includes installation and usage instructions and links to further example data sets. (ZIP) [file pcbi.1008364.s004.zip › pyTFM/docs/images/FEM_area.png]

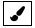

Supplement: S1 Archive — This archive contains the pyTFM source code and documentation which includes installation and usage instructions and links to further example data sets. (ZIP) [file pcbi.1008364.s004.zip › pyTFM/docs/images/brush.png]

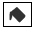

Supplement: S1 Archive — This archive contains the pyTFM source code and documentation which includes installation and usage instructions and links to further example data sets. (ZIP) [file pcbi.1008364.s004.zip › pyTFM/docs/images/bucket.png]

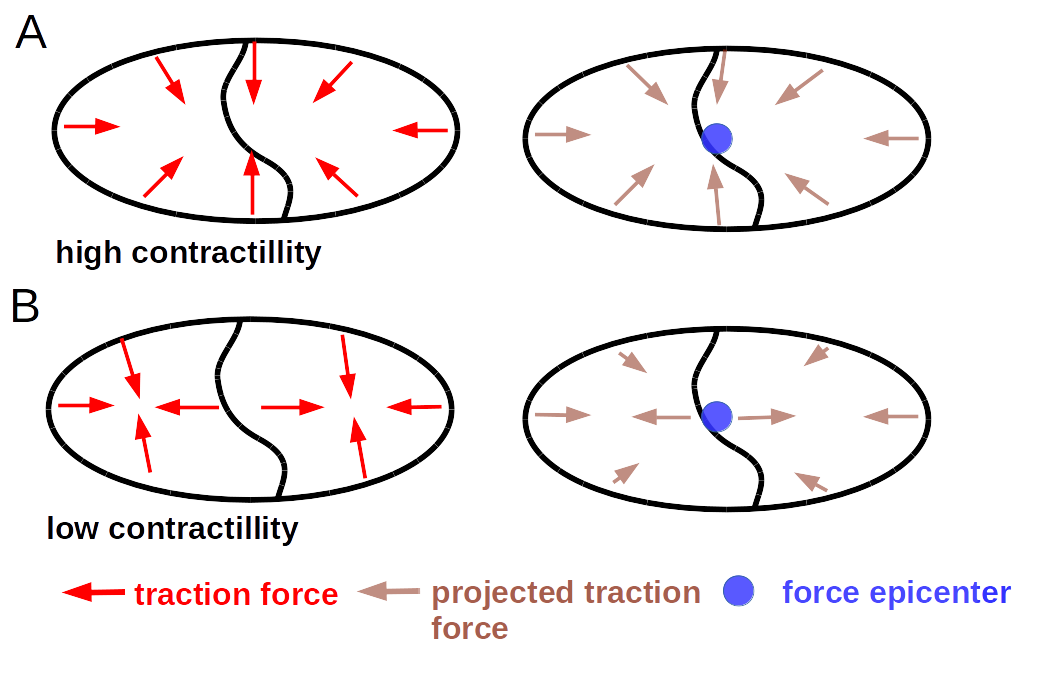

Supplement: S1 Archive — This archive contains the pyTFM source code and documentation which includes installation and usage instructions and links to further example data sets. (ZIP) [file pcbi.1008364.s004.zip › pyTFM/docs/images/contractility.png]

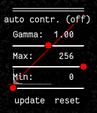

Supplement: S1 Archive — This archive contains the pyTFM source code and documentation which includes installation and usage instructions and links to further example data sets. (ZIP) [file pcbi.1008364.s004.zip › pyTFM/docs/images/control.png]

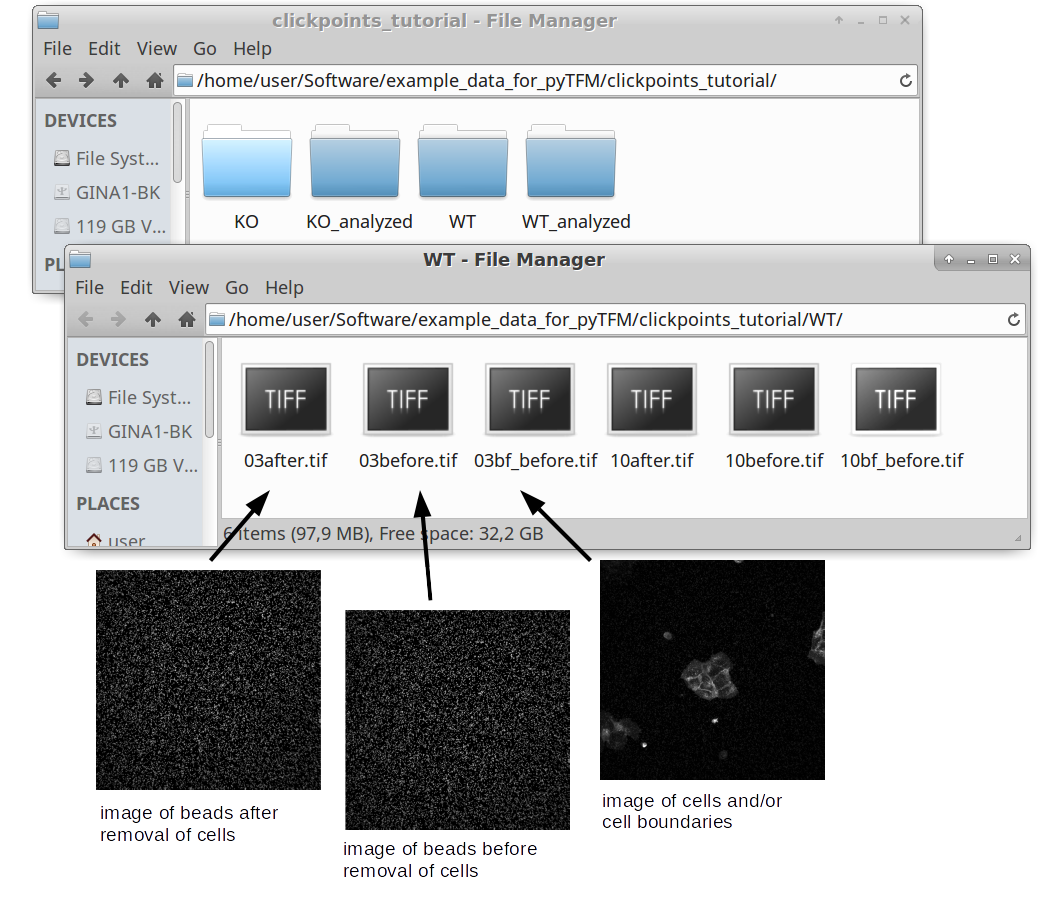

Supplement: S1 Archive — This archive contains the pyTFM source code and documentation which includes installation and usage instructions and links to further example data sets. (ZIP) [file pcbi.1008364.s004.zip › pyTFM/docs/images/data.png]

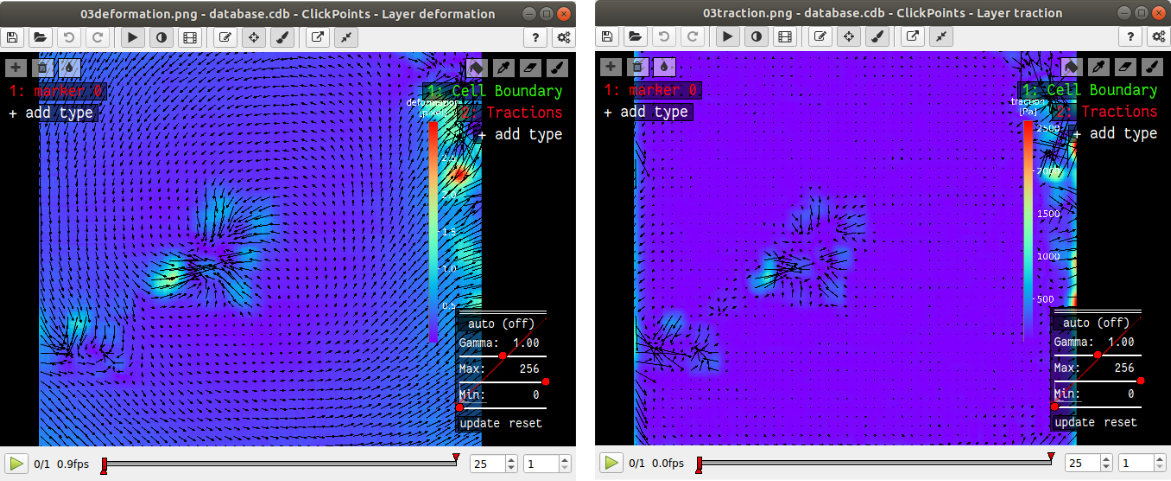

Supplement: S1 Archive — This archive contains the pyTFM source code and documentation which includes installation and usage instructions and links to further example data sets. (ZIP) [file pcbi.1008364.s004.zip › pyTFM/docs/images/def_trac_res.png]

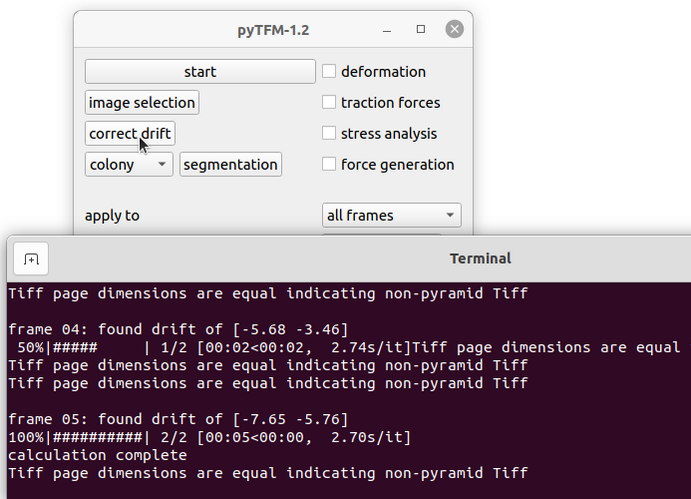

Supplement: S1 Archive — This archive contains the pyTFM source code and documentation which includes installation and usage instructions and links to further example data sets. (ZIP) [file pcbi.1008364.s004.zip › pyTFM/docs/images/drift.png]

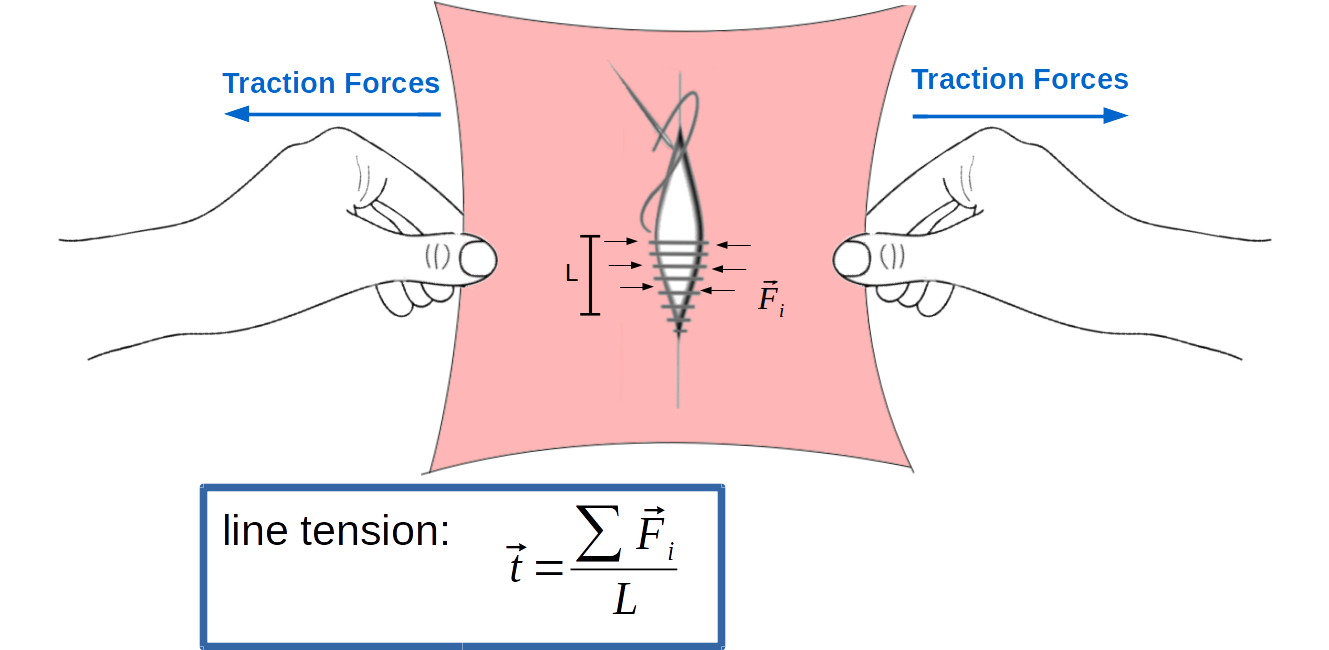

Supplement: S1 Archive — This archive contains the pyTFM source code and documentation which includes installation and usage instructions and links to further example data sets. (ZIP) [file pcbi.1008364.s004.zip › pyTFM/docs/images/line_tension.png]

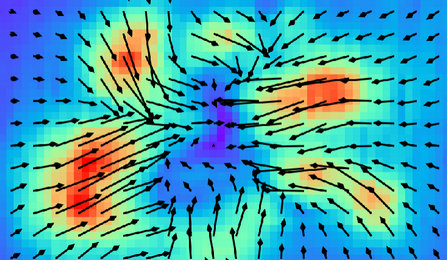

Supplement: S1 Archive — This archive contains the pyTFM source code and documentation which includes installation and usage instructions and links to further example data sets. (ZIP) [file pcbi.1008364.s004.zip › pyTFM/docs/images/logo.png]

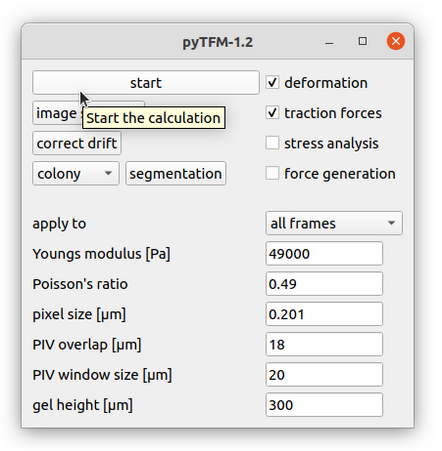

Supplement: S1 Archive — This archive contains the pyTFM source code and documentation which includes installation and usage instructions and links to further example data sets. (ZIP) [file pcbi.1008364.s004.zip › pyTFM/docs/images/main.png]

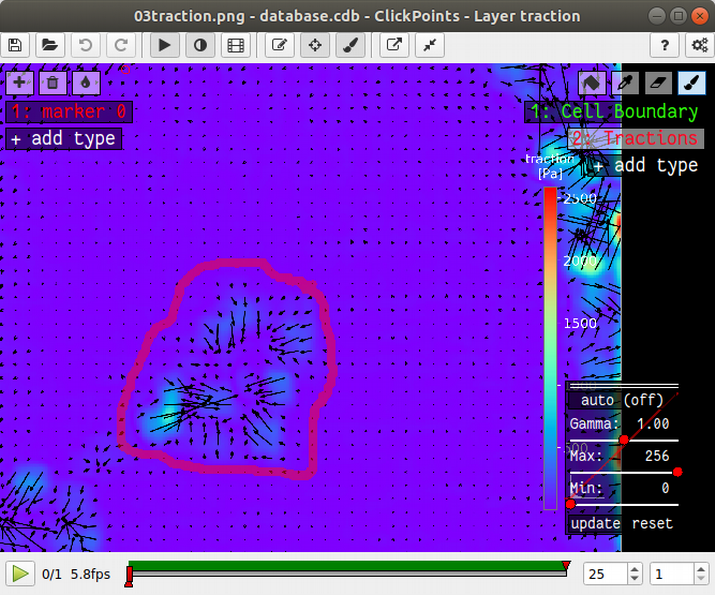

Supplement: S1 Archive — This archive contains the pyTFM source code and documentation which includes installation and usage instructions and links to further example data sets. (ZIP) [file pcbi.1008364.s004.zip › pyTFM/docs/images/mask_force_measures.png]

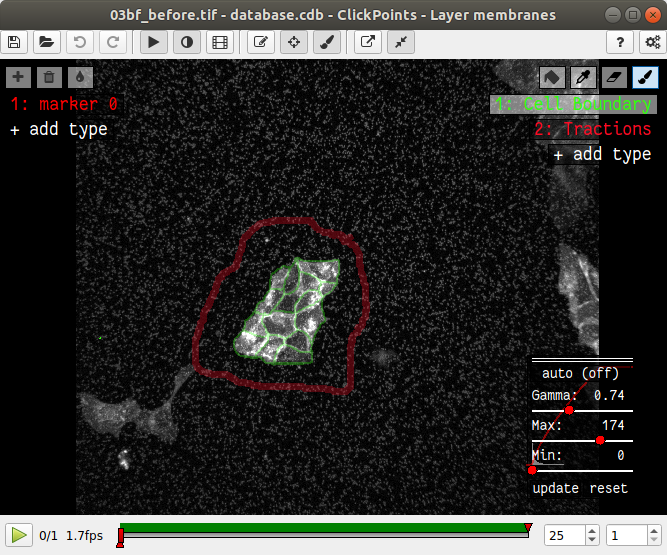

Supplement: S1 Archive — This archive contains the pyTFM source code and documentation which includes installation and usage instructions and links to further example data sets. (ZIP) [file pcbi.1008364.s004.zip › pyTFM/docs/images/membrane.png]

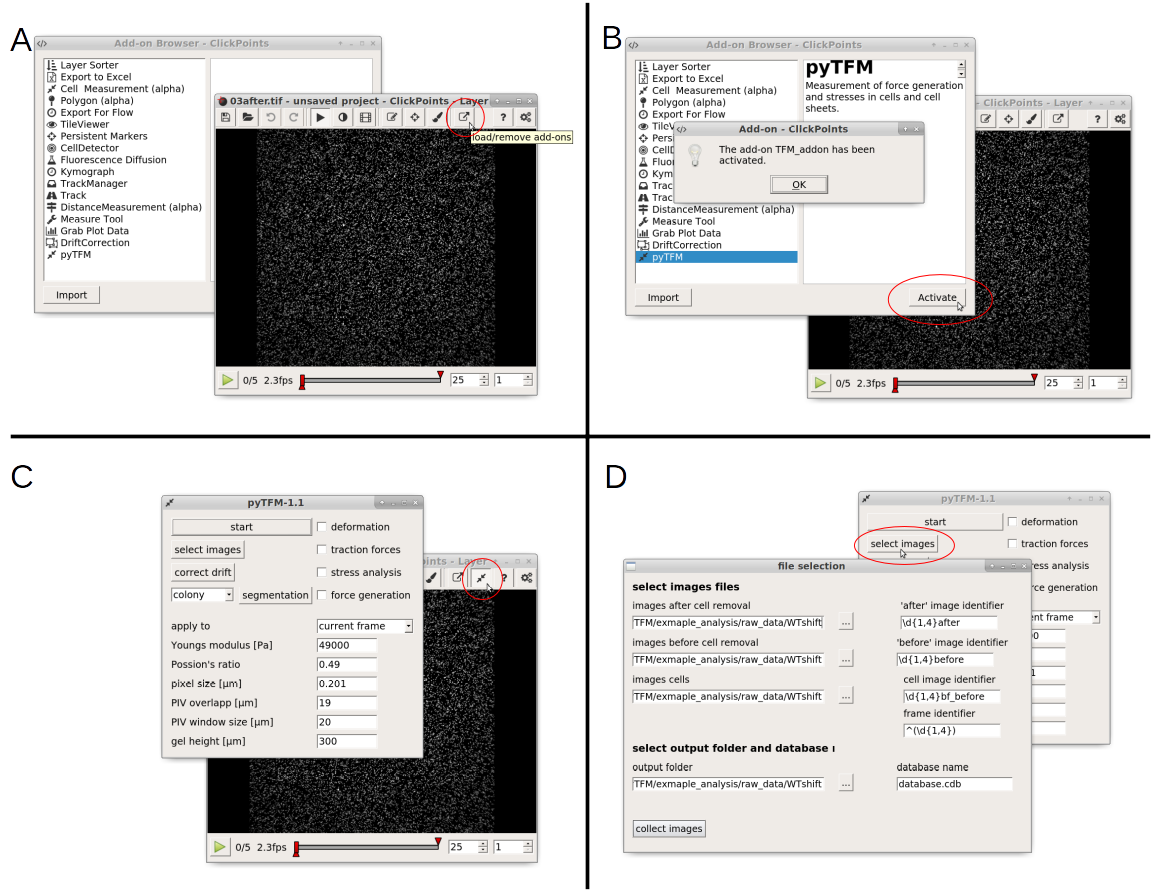

Supplement: S1 Archive — This archive contains the pyTFM source code and documentation which includes installation and usage instructions and links to further example data sets. (ZIP) [file pcbi.1008364.s004.zip › pyTFM/docs/images/open_select_images.png]

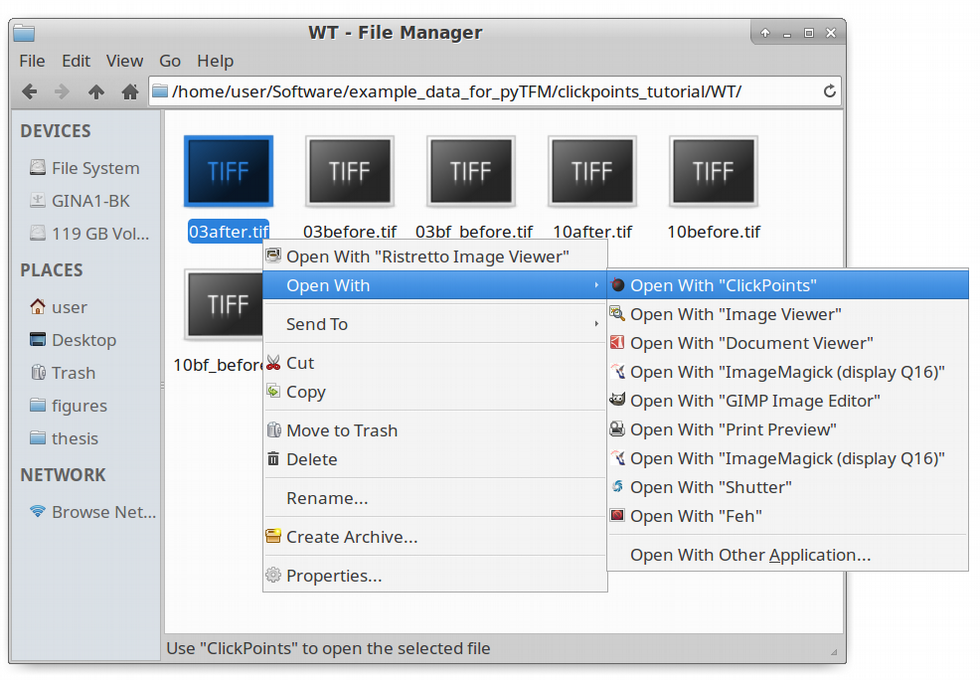

Supplement: S1 Archive — This archive contains the pyTFM source code and documentation which includes installation and usage instructions and links to further example data sets. (ZIP) [file pcbi.1008364.s004.zip › pyTFM/docs/images/open_with_clickpoints.png]

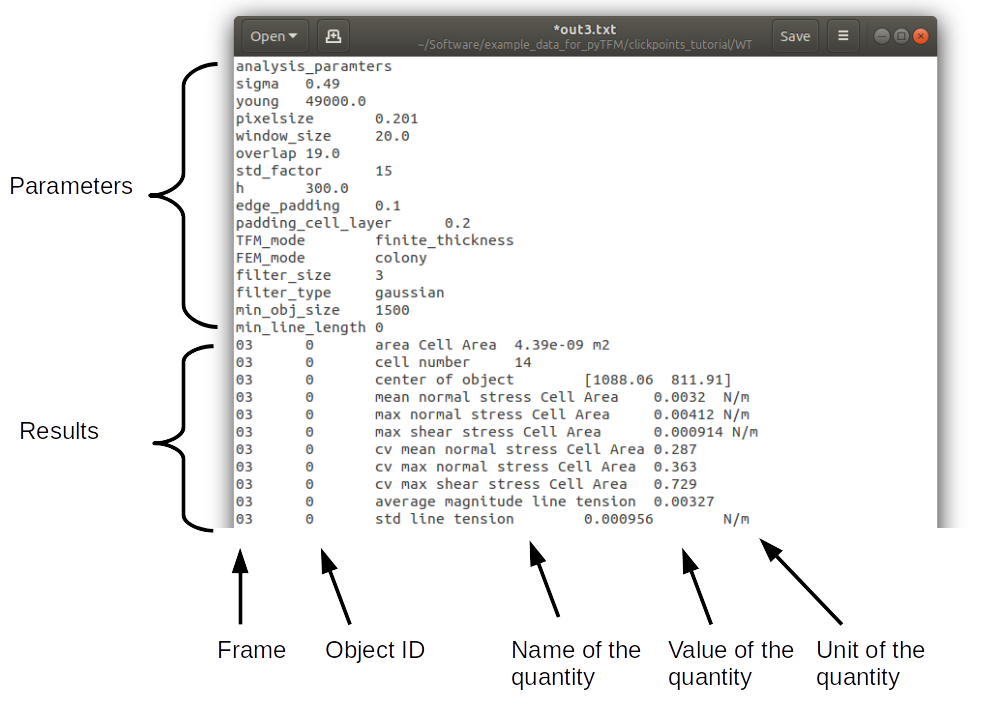

Supplement: S1 Archive — This archive contains the pyTFM source code and documentation which includes installation and usage instructions and links to further example data sets. (ZIP) [file pcbi.1008364.s004.zip › pyTFM/docs/images/out.png]

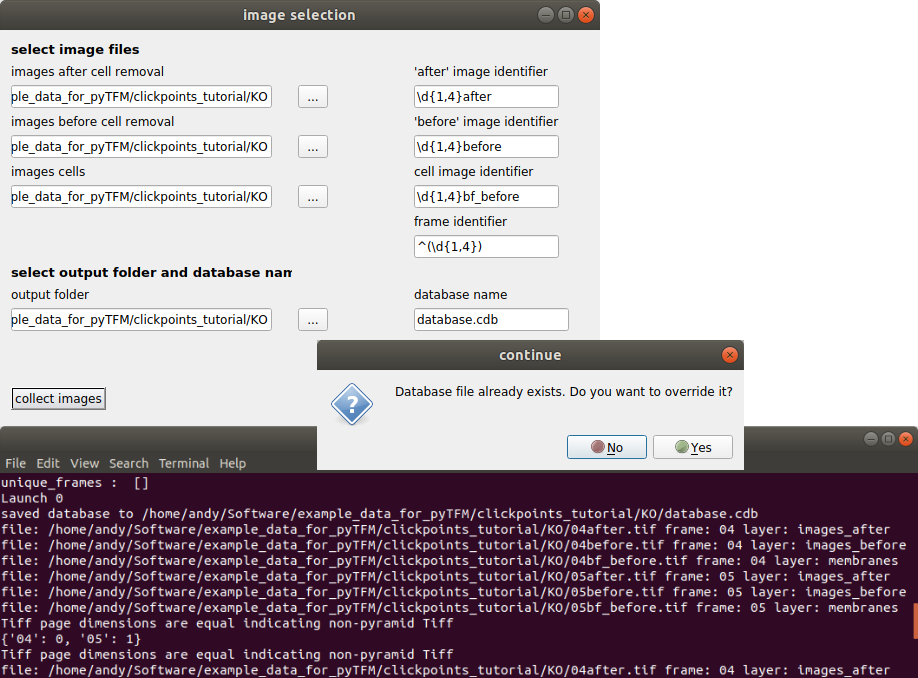

Supplement: S1 Archive — This archive contains the pyTFM source code and documentation which includes installation and usage instructions and links to further example data sets. (ZIP) [file pcbi.1008364.s004.zip › pyTFM/docs/images/output_select_images.png]

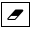

Supplement: S1 Archive — This archive contains the pyTFM source code and documentation which includes installation and usage instructions and links to further example data sets. (ZIP) [file pcbi.1008364.s004.zip › pyTFM/docs/images/rubber.png]

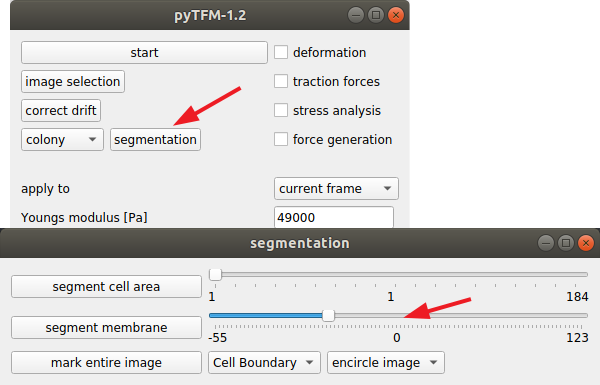

Supplement: S1 Archive — This archive contains the pyTFM source code and documentation which includes installation and usage instructions and links to further example data sets. (ZIP) [file pcbi.1008364.s004.zip › pyTFM/docs/images/segmentation.png]

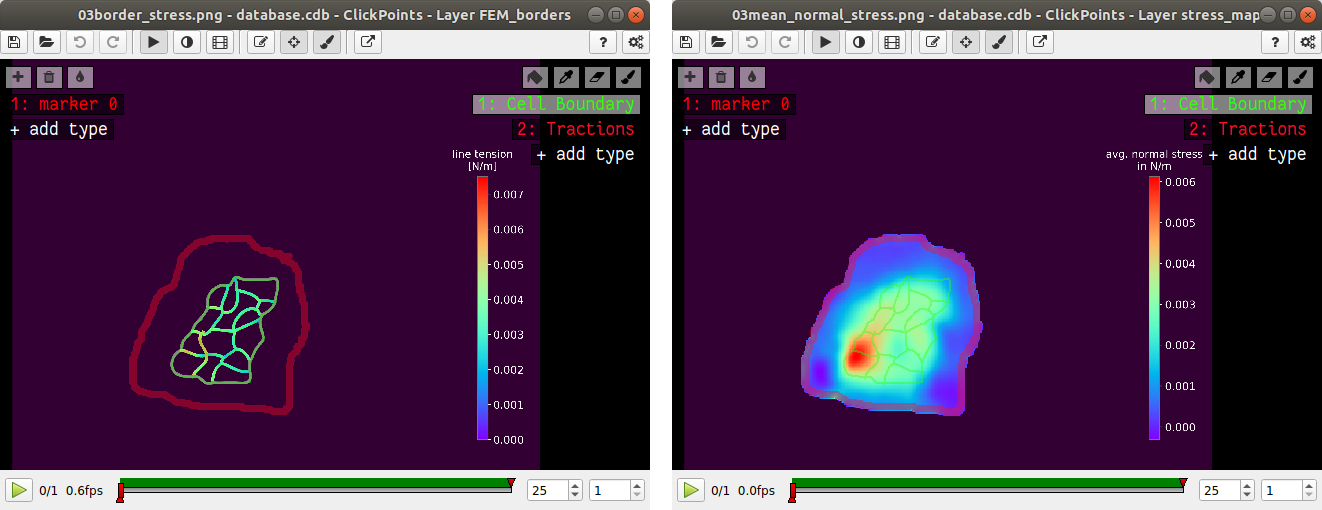

Supplement: S1 Archive — This archive contains the pyTFM source code and documentation which includes installation and usage instructions and links to further example data sets. (ZIP) [file pcbi.1008364.s004.zip › pyTFM/docs/images/stress_res.png]

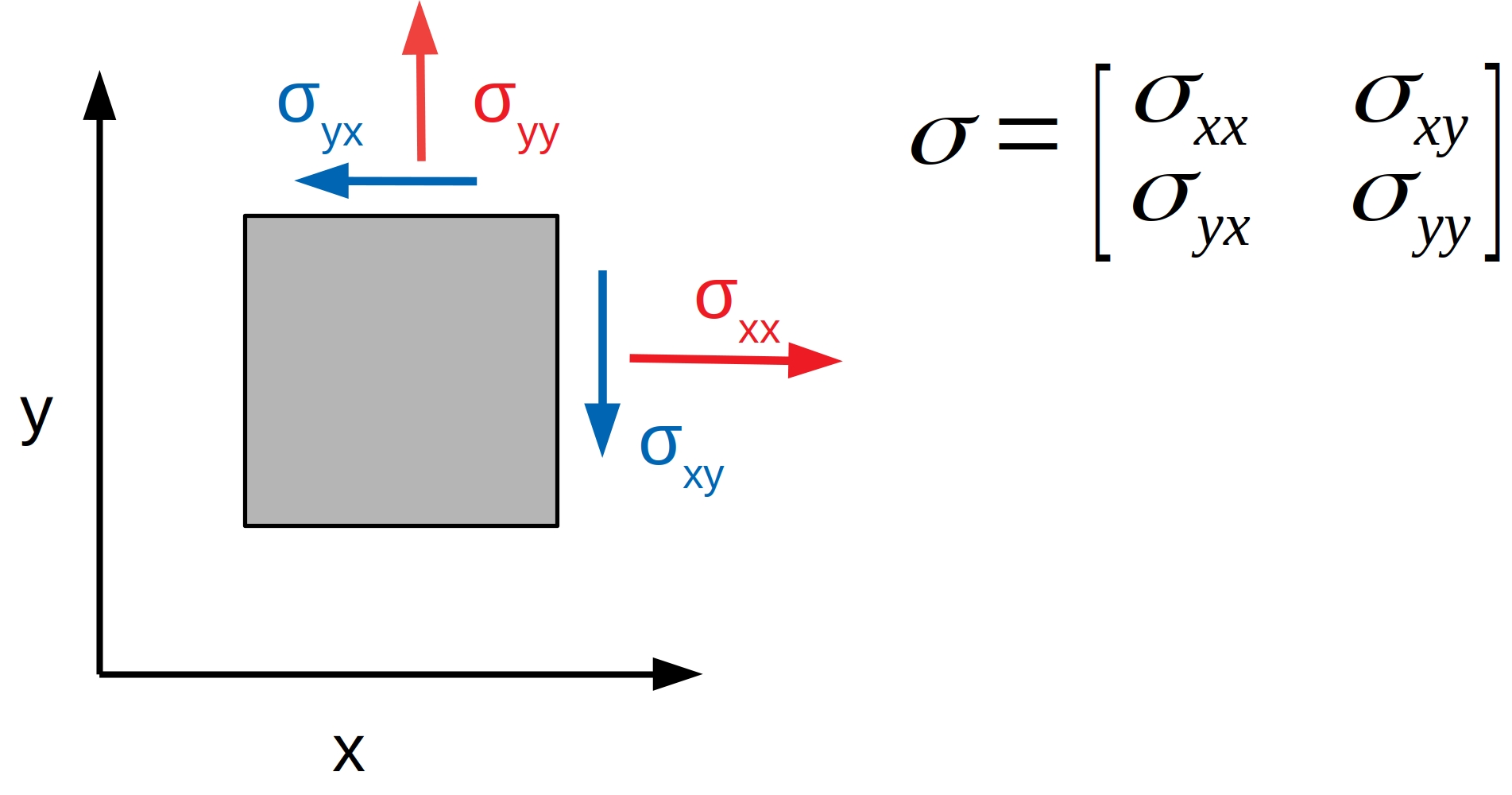

Supplement: S1 Archive — This archive contains the pyTFM source code and documentation which includes installation and usage instructions and links to further example data sets. (ZIP) [file pcbi.1008364.s004.zip › pyTFM/docs/images/stress_tensor.jpg]

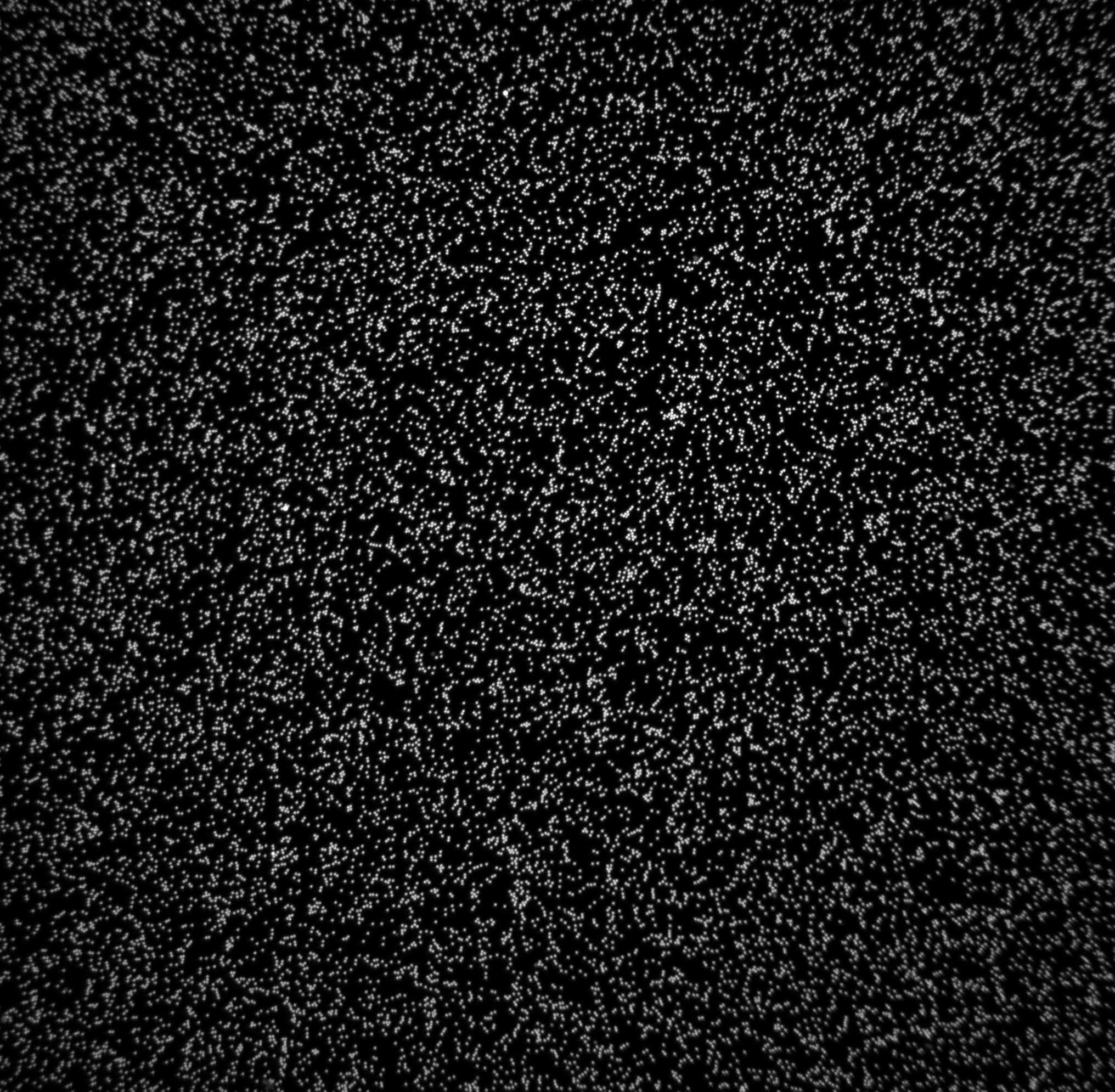

Supplement: S1 Archive — This archive contains the pyTFM source code and documentation which includes installation and usage instructions and links to further example data sets. (ZIP) [file pcbi.1008364.s004.zip › pyTFM/docs/jpeg_example_images/images0.jpg]

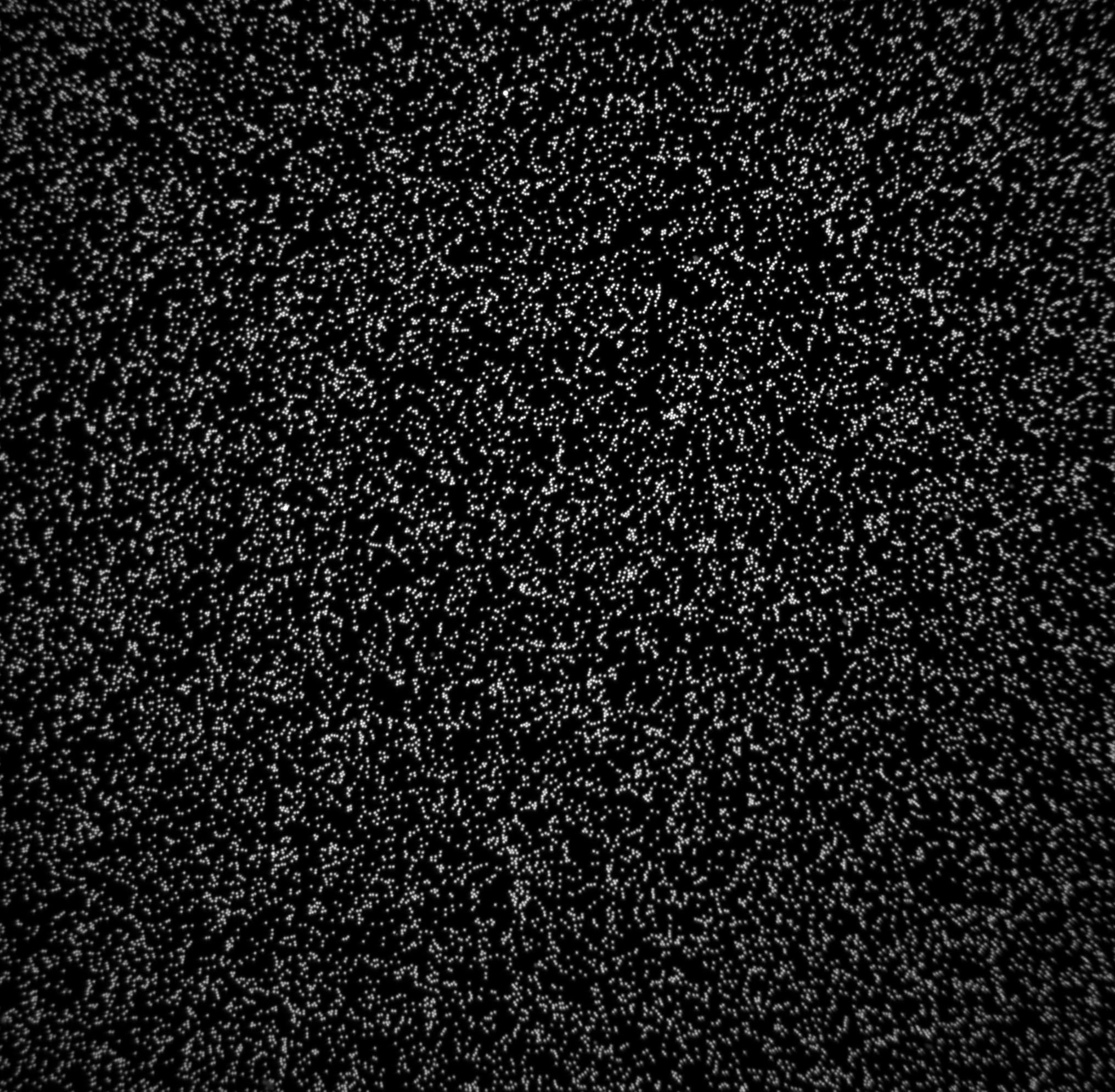

Supplement: S1 Archive — This archive contains the pyTFM source code and documentation which includes installation and usage instructions and links to further example data sets. (ZIP) [file pcbi.1008364.s004.zip › pyTFM/docs/jpeg_example_images/images1.jpg]

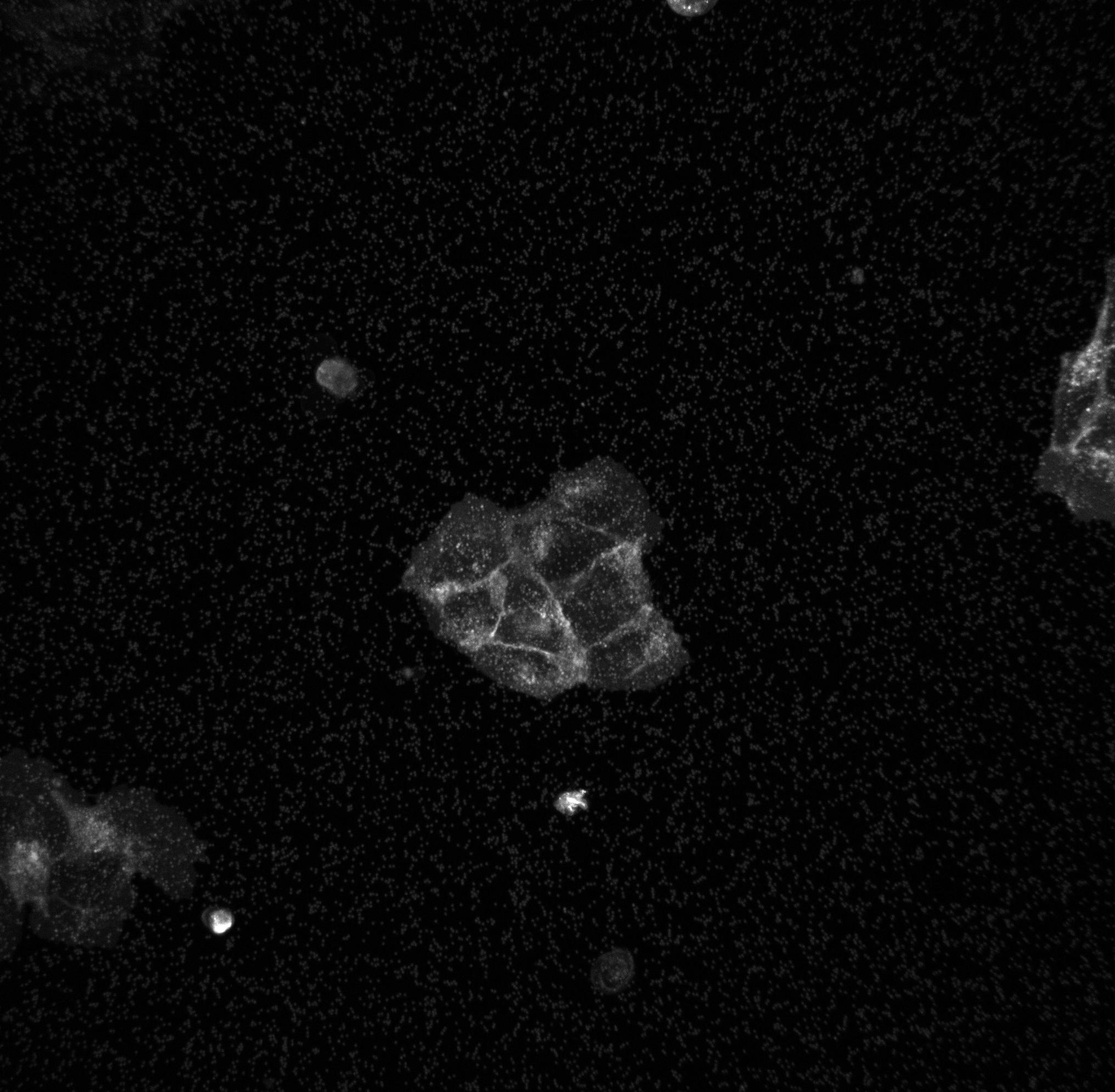

Supplement: S1 Archive — This archive contains the pyTFM source code and documentation which includes installation and usage instructions and links to further example data sets. (ZIP) [file pcbi.1008364.s004.zip › pyTFM/docs/jpeg_example_images/images3.jpg]

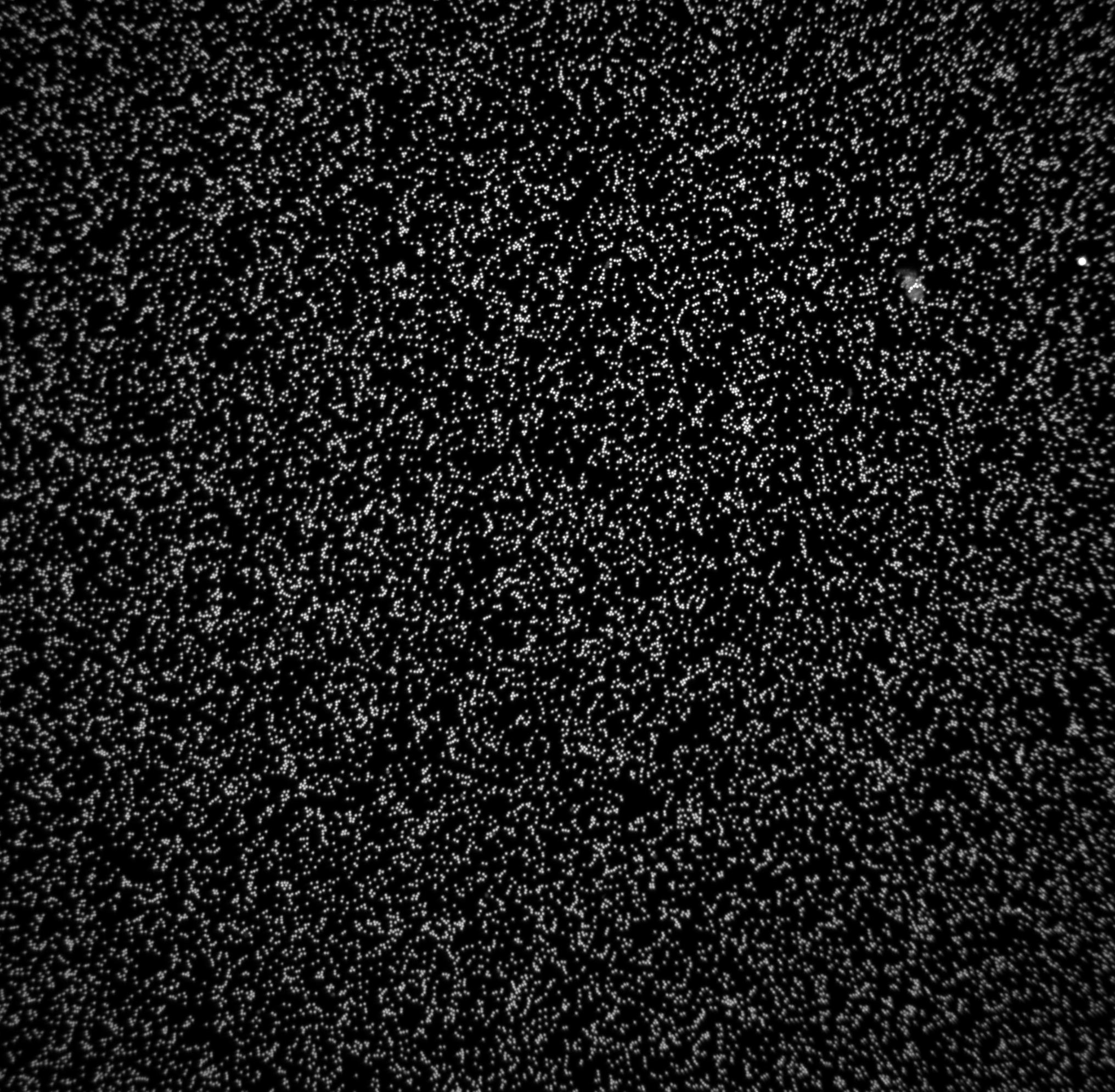

Supplement: S1 Archive — This archive contains the pyTFM source code and documentation which includes installation and usage instructions and links to further example data sets. (ZIP) [file pcbi.1008364.s004.zip › pyTFM/docs/jpeg_example_images/images4.jpg]

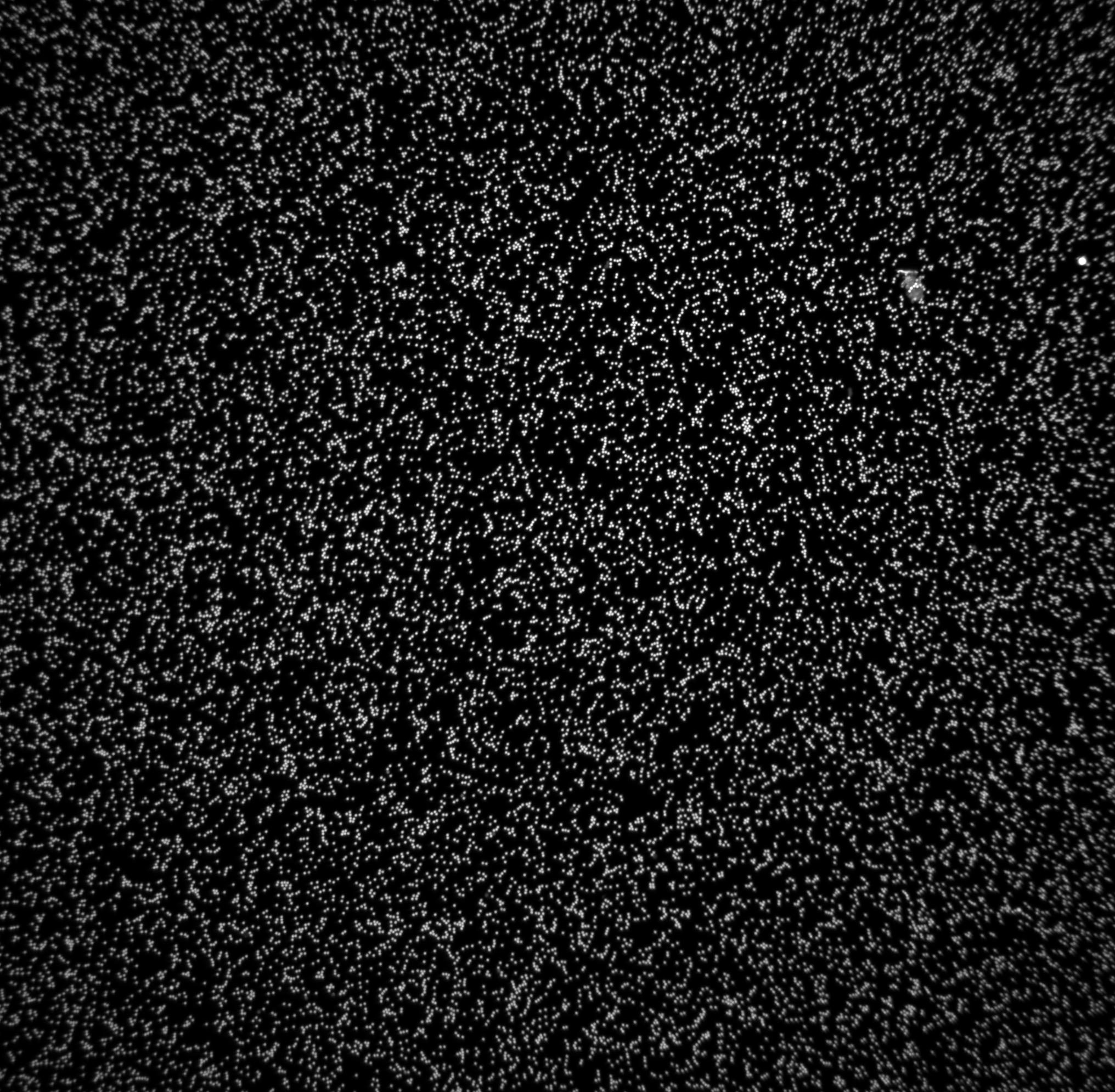

Supplement: S1 Archive — This archive contains the pyTFM source code and documentation which includes installation and usage instructions and links to further example data sets. (ZIP) [file pcbi.1008364.s004.zip › pyTFM/docs/jpeg_example_images/images5.jpg]
