# Supplementary material for: pyTFM: A tool for traction force and monolayer stress microscopy
Source: PLoS Comput Biol. 2021 Jun 21;17(6):e1008364. doi: 10.1371/journal.pcbi.1008364 (PMC8248623; doi:10.1371/journal.pcbi.1008364)
Supplement: Attachment — Submitted filename: PLoS Comput Biol - Review - 2020.pdf [file pcbi.1008364.s005.pdf]

The manuscript presents python-based, well-documented, open-source implementation of traction force microscopy and monolayer stress microscopy. The software developed by the authors, pyTFM, will likely be a very important contribution to the field of cellular mechanics. It will likely attract new research groups to the field and facilitate expansion of our knowledge about the physical biology of the cell.

This contribution will be important particularly because the area of cell mechanics needs a little more uniformity and standardization in the data analysis approaches. But for this manuscript to meet this specific key goal, following inaccuracies must be addressed.

1. Points associated with the theoretical aspects of the work:
    a. Lines 33-27: Refs 16 and 17 do not explicitly “model … point-like contacts to the matrix”,
    b. Lines 47-48: Young’s modulus having categorically “no influence” is true only in the limit of homogeneous elastic properties throughout the monolayer,
    c. Lines 86, 92: Transduction of force is not the same as transmission of force,
    d. Lines 104-109: Incorrect suggestion that Ref 17 does not include analysis for cell patch that does not require exclusion of region close to the image edge,
    e. Lines 206-210: Force/moment balance is not same as displacement/rotation balance,
    f. Lines 231-233: Inhomogeneous is not the same as nonlinear elastic,
    g. Lines 273-305: Common meaning of the term "FEA grid size" is size of individual elements but the manuscript uses this term to mean size of analyzed region. This is particularly important because of point #2 below.
2. Discussion and analysis of the size of individual elements is extremely important, more so in the context of individual cell or small cell patch. But it is entirely missing from the manuscript.
3. Another point that needs more attention is drift correction and its contribution to the accuracy of the results.
4. Description of the effect of extra area outside of the cell island in calculation of monolayer stresses need to include the relationship of this area with the size of the cell patch. Specifically that the results are more sensitive to such an area when the cell patch is small.
