# Supplementary material for: pyTFM: A tool for traction force and monolayer stress microscopy
Source: PLoS Comput Biol. 2021 Jun 21;17(6):e1008364. doi: 10.1371/journal.pcbi.1008364 (PMC8248623; doi:10.1371/journal.pcbi.1008364)
Supplement: Attachment — Submitted filename: Response_to_Reviewers.pdf [file pcbi.1008364.s006.pdf]

FRIEDRICH-ALEXANDER
UNIVERSITÄT
ERLANGEN-NÜRNBERG

Zentrum für Medizinische
Physik und Technik

Department für PhysikLehrstuhl für Biophysik
Andreas Bauer
Henkestraße 91, 91052 Erlangen
Email: andreas.b.bauer@fau.de
http://bio.physik.fau.de

Erlangen, 20. Mar 2020

Manja Marz
Software Editor
PLOS Computational Biology

Dear Prof. Dr. Marz,

Please find enclosed the revised manuscript entitled ***"pyTFM: A tool for Traction Force and Monolayer Stress Microscopy"*** for publication in *PLOS Computational Biology.*

We thank the reviewers for their supportive and valuable comments, and have modified the manuscript to address all points raised by the reviewers (please find below a point-by-point response). Changes to the original manuscript text are marked in green.

Reviewer 1:
We agree with all points that were raised by the reviewer and summarily thank him or her for pointing out our mistakes and errors.

a. Lines 33-27: Refs 16 and 17 do not explicitly "model ... point-like contacts to the matrix"

The reviewer is correct. We changed the sentence as follows:
"The stress tensor field for cells grown in a 2-dimensional environment can be calculated using the Monolayer Stress Microscopy method [16,17], whereby the cell or cell patch is modeled as an elastically stretched 2-dimensional sheet in contact with the matrix so that the external tractions are balanced by the internal stress of the elastic sheet."

b. Lines 47-48: Young's modulus having categorically "no influence" is true only in the limit of homogeneous elastic properties throughout the monolayer

The reviewer is correct. We changed the sentence as follows:

"Consequently, in the limit of homogeneous elastic properties throughout the cell sheet, its Young's modulus has no influence on the stress estimation, and its Poisson's ratio has only a negligible influence."

c. Lines 86, 92: Transduction of force is not the same as transmission of force, and line 105, 109:

Throughout the text we are now using the term force transmission.

d. Lines 104-109: Incorrect suggestion that Ref 17 does not include analysis for cell patch that does not require exclusion of region close to the image edge

We assume that the reviewer refers to lines 197-201. In the revised manuscript, we do no longer refer to Ref 17 and only explain the strategy employed in pyTFM for constraining the solution regarding translation and rotation:

"Eq. 4 is not uniquely solvable because rigid rotations and displacements of the whole system are not constrained. pyTFM addresses this problem by modifying Eq. 4 as follows:"

e. Lines 206-210: Force/moment balance is not same as displacement/rotation balance

Equation 6 was actually incorrect. The correct equation (which was always correctly implemented in pyTFM) and modified text is:

"First, to ensure that all forces and torques of the cell or cell patch are balanced, the forces applied to the FEM-grid are corrected by subtracting the net force and by rotating all force vectors to enforce zero torque. Second, zero global displacement and zero global rotation conditions are imposed:

$$\sum(d_x) = 0$$

$$\sum(d_y) = 0 \qquad (6)$$

$$\sum(d_x r_y - d_y r_x) = 0$$

rx and ry are the components of the distance vector of the corresponding node to the center of the FEM-grid. These constraints are directly added to the system of equations in equation 4. The combined system of Eqs. 6 and Eq. 4 is solved numerically using a standard least-squares minimization."

f. Lines 231-233: Inhomogeneous is not the same as nonlinear elastic

We wrote "uniform elastic properties" but we actually meant "homogeneous and isotropic elastic properties". The following sentence was also confusing. This has now been corrected:

"For Monolayer Stress Microscopy, cells are modeled as a linearly elastic material with homogeneous and isotropic elastic properties. Although many cell types have been shown to exhibit stress stiffening (the Young's modulus increases with the cellular stress [29]), this has only a small effect on the stresses recovered by Monolayer Stress Microscopy [17]. Furthermore, Monolayer Stress Microscopy models the cells as a 2-dimensional flat sheet. Deviations from this assumption can introduce an error to the stress calculation on the order of $(h/l)^2$ [17], where *h* is the cell height and *l* is the wavelength of the tractions in Fourier-space (in the case of a single cell that generate tractions in the form of two opposing force monopoles, *l* corresponds to the distance between the force monopoles). This error can become relevant for isolated round cells but not for larger flat cell colonies."

g. Lines 273-305: Common meaning of the term "FEA grid size" is size of individual elements but the manuscript uses this term to mean size of analyzed region. This is particularly important because of point #2 below.

In the revised manuscript, we now use the term "FEM-grid area" when we refer to the size of the analyzed region.

2. Discussion and analysis of the size of individual elements is extremely important, more so in the context of individual cell or small cell patch. But it is entirely missing from the manuscript.

To address this point, we calculated the stresses within a MDCK cell colony for different FEM-element sizes. We found that the calculated stresses are not influenced by the FEM-element size below an edge length of the quadrilateral FEM-elements of 3 µm, but calculated stresses decreased for larger FEM-element sizes. We added a discussion and a new figure (Fig S1) to the Supplementary Information.

In practice, the FEM-element size is determined by the Particle Image Velocimetry parameters “PIV overlap” and “PIV windowsize”, which are chosen by the user based on the specific experimental setup. More detailed instructions on choosing these parameters are given in the pyTFM documentation. The default parameters of pyTFM correspond to an element edge length of 1 µm, for which the calculated stresses have reached a stable plateau.

3. Another point that needs more attention is drift correction and its contribution to the accuracy of the results.

In our experience, drift correction is essential for the calculation of deformation fields and thus all following steps. We added the following sentences to the section “Deformation Fields and TFM” :

“A common issue when calculating the deformation field is a global drift of the images, which needs to be corrected prior to PIV. This drift can reach several µm and is caused by the combined effect of positioning inaccuracies of the motorized x,y-stage, mechanical handling or shaking during the addition of trypsin-EDTA, and slow temporal or temperature-induced mechanical drift.
pyTFM offers a global drift correction that works in three steps: First, the drift is estimated with sub-pixel accuracy by cross-correlating the entire first image with the entire second image. This is done with the “phase_cross_correlation” from the *scikit-image*[22] python package. Next, the first image is shifted by the drift, and finally both images are cropped to the overlapping field of view.”

4. Description of the effect of extra area outside of the cell island in calculation of monolayer stresses need to include the relationship of this area with the size of the cell patch. Specifically that the results are more sensitive to such an area when the cell patch is small.

We addressed this point by evaluating the stress recovery using synthetic data, where the cell patch is represented by a square-shaped area subjected to a uniform normal stress. First, we calculated the traction and deformation fields corresponding to the input stress field and then recovered the original stresses according to the workflow described in section 3.1. "Accuracy of TFM and MSM algorithms". We repeated this analysis for various sizes of the cell patch and found that the stresses are insensitive to the cell patch size for an edge length of 50 µm or more. Our approach of expanding the FEM-grid beyond the cell patch always resulted in an improved stress recovery. We have added this result to the section "Effect of FEM-grid area on Stress Recovery", and we appended Fig.4 with Fig. 4c. This results shows that pyTFM is not adequate to calculate stresses in small single cells as had been indicated in the original manuscript. We have removed these indications.

Reviewer 2:

Minor: Page 2, Abstract 3 lines from bottom. This is redundant.

We agree with the reviewer, removed the redundant line and reformulated it to:

"In this work, we also thoroughly analyze the accuracy and performance of Traction Force Microscopy and Monolayer Stress Microscopy algorithms of pyTFM using synthetic and experimental data from epithelial cell patches."
