# Supplementary material for: pyTFM: A tool for traction force and monolayer stress microscopy
Source: PLoS Comput Biol. 2021 Jun 21;17(6):e1008364. doi: 10.1371/journal.pcbi.1008364 (PMC8248623; doi:10.1371/journal.pcbi.1008364)
Supplement: Attachment — Submitted filename: ResponseToReviewers.pdf [file pcbi.1008364.s007.pdf]

FRIEDRICH-ALEXANDER
UNIVERSITÄT
ERLANGEN-NÜRNBERG
Zentrum für Medizinische
Physik und Technik

Department für PhysikLehrstuhl für Biophysik
Andreas Bauer
Henkestraße 91, 91052 Erlangen
Email: andreas.b.bauer@fau.de
https://bio.physik.fau.de

Erlangen, 08. Mar 2021

Manja Marz
Software Editor
PLOS Computational Biology

Dear Prof. Dr. Marz,

Please find enclosed the revised manuscript entitled ***"pyTFM: A tool for Traction Force and Monolayer Stress Microscopy"*** for publication in *PLOS Computational Biology.*

We thank the reviewers for their supportive and valuable comments, and have modified the manuscript to address all points raised by the reviewers (please find below a point-by-point response). Changes to the original manuscript text are marked in light green. Please note that we added two authors, Ingo Thievessen and Lena Fischer, who developed the TD-tomato-farnesyl expressing fibroblasts that were used in the cell experiments. It was an oversight that they were not listed as authors in our first submission.

Reviewer 1:

Reviewer 1 raised several issues regarding the description of our adaptation of the Finite Elements method for small cell patches (lines 204 to 213). We have reformulated the entire paragraph to make our description clearer. In particular in response to the first point raised:

1. "Equation 4 is not uniquely solvable …" - As written the sentence is misleading. The equation is solvable. It is inaccuracies in the measurement of displacement that cause problems. Hence, the sentence must start with the qualifier that "In case of a cell patch, measured displacements can have a nonzero mean translation and rotation. In such case, "

We now specify (see full paragraph below) that Equation 4 is solvable if constraints on nodal deformations are defined.

Updated text does not acknowledge the fact that such analysis of a cell patch has been conducted in reference #17.

We now outline (see below) how this issue (of a unique solution for Eq. 4) was solved in reference #17 (Tambe et. al. 2013).

3. First, to ensure … corrected by subtracting the net force by rotating all force vectors to enforce zero torque" - the meaning of the last part of the sentence is unclear. What is subtracted?

We correct net forces by subtracting the sum of all nodal force vectors from each nodal force vector and correct torques by rotating each nodal force vector by a small angle. This is now better explained, see below

4. One cannot apply both force and displacement constraint on a node. The assertion "Second, zero …" is likely misstated.

Yes, thank you. First, forces and torques of the entire system are corrected and then global zero displacement and rotation conditions are implemented as described below.

The new paragraphs read as follows:

"The FEM algorithm assumes that there are no torques or net forces acting on the cell patch. This must also be true in reality as the cell patch would not be stationary otherwise. However, the TFM algorithm only ensures that forces and torques are globally balanced

(across the entire image), but not necessarily across a cell patch. These unbalanced net forces and torques acting on a cell patch must be corrected prior to performing the FEM algorithm to accurately compute the cellular stresses. pyTFM corrects unbalanced net forces by subtracting the sum of all force vectors of the FEM system from the force vector at each node. The unbalanced net torque is corrected by rotating the direction of all force vectors by a small angle (typically below 5°) until the torque of the entire system is zero.

By constraining the FEM system to zero rigid rotational or translational movement, Eq. 4 is uniquely solvable [29]. These constraints can be applied in two ways: The first option is to apply a boundary condition of zero displacement in x- and y- direction to an arbitrarily chosen node of the FEM grid, and a boundary condition of zero rotation between this fixed node and another arbitrarily chosen second node [17]. In practise, this is implemented by selecting a second node with the same y-coordinate as the fixed node and applying a zero x-displacement boundary condition. The second option, which is implemented in pyTFM, is as follows: Instead of subjecting individual nodes to displacement boundary conditions, we formulate zero rigid displacement and rotation conditions on the whole system in three separate equations (Eq. 6-8), add them to the system of equations in Eq. 4 and finally solve the combined system numerically using a standard least-squares minimization. Eq. 6 and 7 ensure that the sum of all nodal displacements in x- and y-direction is zero, and Eq. 8 ensures that the rotation of all nodes around the center of mass of the FEM system is zero.

$$\Sigma(d_x) = 0 \tag{6}$$

$$\Sigma(d_y) = 0 \tag{7}$$

$$\Sigma(d_x r_y - d_y r_x) = 0 \tag{8}$$

r_x and r_y are the components of the distance vector of the corresponding node to the center of mass of the FEM-grid.”

Reviewer 3:

The reviewer pointed out that not all data for the figures has been provided. We added a spreadsheet (Supplementary file S3) that contains all data points plotted in Figure 4. Note that the raw images of cells and the substrate in tensed and relaxed states (used for the analysis in Fig. 4B, Fig. 5 and Fig. S3) are currently hosted at https://zenodo.org/record/4047040 as was stated in the data availability statement.

Furthermore, reviewer 3 encouraged us to discuss common regularization methods of traction force microscopy algorithms and compare them to the pyTFM traction force microscopy algorithm.

We have added a references to the introduction and now explicitly mention Particle Tracking Velocimetry and Boundary Elements Methods, both of which are relevant for the second point raised by reviewer 3 (see below):

“Typically, the substrate is imaged in a tensed and a relaxed (force-free) state, whereby force relaxation is achieved by detaching the cells from the substrate. These two images are then compared to quantify substrate deformations, either by Particle Tracking Velocimetry (PTV) where individual marker beads are tracked, or by cross-correlation based Particle Image Velocimetry (PIV) [9].”

“The calculation of the traction field from the deformation field is an inverse problem for which a number of algorithms have been developed, including numerical methods such as the Boundary Elements Method [11, 12], Fourier-based deconvolution, and Finite Element (FE) computations, all of which have specific advantages and disadvantages (see [15] and [12] for a detailed discussion). pyTFM uses the Fourier Transform Traction Cytometry (FTTC) algorithm [13], as it is computationally fast and does not require knowledge of the cell boundary.“

Furthermore, in section *Design and Implementation,* subsection *Deformation fields and TFM*, we added the following paragraph:

“One particular challenge of Traction Force Microscopy is that noise in the deformation field can lead to large errors in the traction field. This can be remedied by regularization of the reconstructed forces, e.g. by adding the L1 or L2 norm to the cost function of the inverse minimization problem [23]. By contrast, pyTFM does not use explicit regularization but instead smooths the calculated traction field with a user-defined Gaussian low-pass filter, with a sigma of typically 3 µm. This effectively suppresses all tractions with high spatial frequencies but inherently limits the spatial resolution of the pyTFM algorithm. The appropriate degree of smoothing depends on the spatial resolution of the deformation field, which in turn depends e.g. on the density of fiducial markers, the window size for the PIV algorithm or image noise. The user is encouraged to test different values for sigma and to

select the smallest value for which the noise in the cell-free areas is still tolerable in comparison to the magnitude of cell tractions.”

We acknowledge that we treat the difficult problem of regularization or smoothing in our manuscript only superficially. This is partially because we have not implemented L1 or L2 regularization in pyTFM, partially because there is currently little consensus on how to choose the smoothing parameter objectively, as the traction field output depends not only on noise but also the PIV algorithm, window size, or the density of marker beads. The standard L-curve method for choosing the regularization parameter may fail in the presence of too much or ”patterned” noise, as pointed out by Huang et. al. (Sci. Rep. 2019). We have also not discussed the subtle difference between regularization and smoothing, and believe that this important topic should be dealt with in a separate, dedicated manuscript.

Reviewer 3 also advised us to list alternative Traction Force Microscopy software and point to their specific advantages. We have done so by adding the following to the Section *Availability and future directions*:

“pyTFM provides a user-friendly implementation of Traction Force Microscopy and Monolayer Stress Microscopy in a combined image and data analysis pipeline. For users interested only in Traction Force Microscopy, several other intuitive software packages are freely available.

The FTTC and PIV ImageJ plugins [22], hosted at https://sites.google.com/site/qingzongtseng/tfm#publications, can analyze the typical traction force experiment in which the substrate is imaged once before and once after force relaxation. The software makes use of the ImageJ framework to organize input images and output plots. It calculates deformation fields using the standard PIV algorithm and traction fields using the L2-regularized FTTC algorithm. The deformation field can be filtered with a number of methods. Additionally, this plugin can calculate the strain energy over a user-selected area.

The TFM MATLAB package [23], hosted at https://github.com/DanuserLab/TFM, uses PIV or PTV to calculate the deformation field. Traction fields can be calculated either with the L1- or L2-regularized FTTC algorithm or the L2-regularized Boundary Elements Method. Appropriate regularization parameters can be selected using the L-curve method [23]. This package also allows for the analysis of TFM-experiments where the evolution of cellular forces is measured over time.

Another MATLAB tool [24], hosted at https://data.mendeley.com/datasets/229bnpp8rb/1, implements Bayesian FTTC [12], thus providing a method to automatically select the regularization parameter. Additionally, this package can also perform traditional L2-regularized FTTC and enables the user to manually select the regularization parameter using the L-curve method. However, the user needs to provide the deformation field as an input.”
